# Semi-automated Segmentation and Quantification of Perivascular Spaces at 7 Tesla in COVID-19

**DOI:** 10.3389/fneur.2022.846957

**Published:** 2022-04-01

**Authors:** Mackenzie T. Langan, Derek A. Smith, Gaurav Verma, Oleksandr Khegai, Sera Saju, Shams Rashid, Daniel Ranti, Matthew Markowitz, Puneet Belani, Nathalie Jette, Brian Mathew, Jonathan Goldstein, Claudia F. E. Kirsch, Laurel S. Morris, Jacqueline H. Becker, Bradley N. Delman, Priti Balchandani

**Affiliations:** ^1^Icahn School of Medicine at Mount Sinai, New York, NY, United States; ^2^Biomedical Engineering and Imaging Institute at Mount Sinai School of Medicine, New York, NY, United States; ^3^The Graduate Center, City University of New York, New York, NY, United States; ^4^Department of Diagnostic, Molecular and Interventional Radiology, Icahn School of Medicine at Mount Sinai, New York, NY, United States; ^5^Department of Neurology, Icahn School of Medicine at Mount Sinai, New York, NY, United States; ^6^Department of Population Health Science and Policy, Icahn School of Medicine at Mount Sinai, New York, NY, United States; ^7^Department of Radiology, Zucker Hofstra School of Medicine at Northwell Health, Uniondale, NY, United States; ^8^Department of Psychiatry at the Icahn School of Medicine at Mount Sinai, New York, NY, United States; ^9^Division of General Internal Medicine, Icahn School of Medicine at Mount Sinai, New York, NY, United States; ^10^Nash Family Department of Neuroscience, Icahn School of Medicine at Mount Sinai, New York, NY, United States

**Keywords:** Virchow Robin spaces, coronavirus, neuroinflammation, Frangi filter, 7 T MRI, semiautomated

## Abstract

While COVID-19 is primarily considered a respiratory disease, it has been shown to affect the central nervous system. Mounting evidence shows that COVID-19 is associated with neurological complications as well as effects thought to be related to neuroinflammatory processes. Due to the novelty of COVID-19, there is a need to better understand the possible long-term effects it may have on patients, particularly linkage to neuroinflammatory processes. Perivascular spaces (PVS) are small fluid-filled spaces in the brain that appear on MRI scans near blood vessels and are believed to play a role in modulation of the immune response, leukocyte trafficking, and glymphatic drainage. Some studies have suggested that increased number or presence of PVS could be considered a marker of increased blood-brain barrier permeability or dysfunction and may be involved in or precede cascades leading to neuroinflammatory processes. Due to their size, PVS are better detected on MRI at ultrahigh magnetic field strengths such as 7 Tesla, with improved sensitivity and resolution to quantify both concentration and size. As such, the objective of this prospective study was to leverage a semi-automated detection tool to identify and quantify differences in perivascular spaces between a group of 10 COVID-19 patients and a similar subset of controls to determine whether PVS might be biomarkers of COVID-19-mediated neuroinflammation. Results demonstrate a detectable difference in neuroinflammatory measures in the patient group compared to controls. PVS count and white matter volume were significantly different in the patient group compared to controls, yet there was no significant association between PVS count and symptom measures. Our findings suggest that the PVS count may be a viable marker for neuroinflammation in COVID-19, and other diseases which may be linked to neuroinflammatory processes.

## Introduction

While COVID-19 is primarily considered a respiratory disease caused by the SARS-CoV-2 virus (1), numerous studies have demonstrated involvement of the central nervous system (CNS) (2). While infection rates have risen since the inception of the pandemic in 2020, there has been a secondary epidemic among patients who have recovered from COVID-19 presenting with persistent neurological manifestations. These patients suffer from a diverse array of symptoms that persist long after viral resolution of primary disease, frequently including neurological manifestations such as fatigue, brain fog or other cognitive dysfunction, headaches, encephalopathy, anosmia, and anxiety, among others (2–6). The mounting evidence that long haul COVID-19 is associated with chronic neurological complications suggests that these neurological processes may be a byproduct of or related to neuroinflammatory processes, possibly caused by disruption to neuronal networks and metabolic homeostasis (7). As of February of 2022, over 430 million people had been infected with SARS-CoV-2, and up to one-third of survivors are at risk for long haul COVID with neurological sequelae (8). As the number of COVID-19 survivors increases, there is a critical need to better understand the pathologic neuroinflammatory effects that COVID-19 has on the central nervous system.

Numerous case reports and emerging case series have linked inflammatory processes and CNS complications to COVID-19 pathology and disease progression (9). Neurological impairments in long haul COVID have been attributed to neuroinvasion, hyper-neuroinflammation, blood brain barrier (BBB) disruption and dysfunction, and possibly provoked by impairments caused by maladaptive innate immunity (2, 6). The RAS-mediated bradykinin storm has been explored, providing a possible explanation for the appearance of neurological symptoms linked to inflammatory responses (10). The overall mediation of neuroinflammation in COVID-19 may be due to cytokine or bradykinin storm. It has been postulated that blood brain barrier disruption with concomitant increased permeability results from pro-inflammatory cytokines such as IL-1β, IL-6, IL-17, and TNFα, which in turn activate glial cells with microglial proliferative changes. IL-17 upregulates the pro-inflammatory cytokines IL-1β, IL-6, and TNFα, the chemokines CCL2 and MIP-2/IL-8 and pro-inflammatory cyclooxygenase-2, prostaglandin E2 and nitric oxide (6). The increase in IL-17 overwhelms T-cells, leading to their cell death through apoptosis and a resulting lymphopenia, and leads to inflammation from nuclear factor kappa-light enhanced activated B-cells (NKκB). Both processes lead to increased neurotoxicity (6).

Magnetic resonance imaging (MRI) is a valuable tool in the characterization of neuropsychiatric and neurological disorders, with ultra-high field (UHF) imaging offering improved signal to noise ratios and higher spatial and contrast resolution over conventional high-field strengths (11–15). The detail and increased resolution achieved at 7 Tesla (7 T) has enhanced the *in vivo* study of neurodegenerative conditions through improved detection of microstructural differences, including perivascular spaces (PVS), above what is achievable at lower field strengths (16–20).

Perivascular spaces are small fluid-containing spaces surrounding blood vessels within the brain. While these may be seen as a normal function of aging, they are also believed to play a role in modulation of the immune response, leukocyte trafficking, and glymphatic drainage. Their presence is linked to a broad range of neurological conditions such as dementia, multiple sclerosis, epilepsy, pseudotumor cerebri, traumatic brain injury, and cerebral small vessel disease (21–28). Prominence of PVS is also strongly associated with the development and progression of neurological disease (29, 30). Research has suggested that increased quantity of PVS may be a potential biomarker of increased blood brain barrier (BBB) permeability or dysfunction and a marker for processes linked to cascades leading to neuroinflammation (20, 31). Additionally, increased number of PVS is linked to dysfunction of the glymphatic clearance system causing a reduction in metabolic waste removal (32). Accumulation of these cellular waste products could contribute to the cascade of neuroinflammatory processes and ultimately contribute to the development or exacerbation of neurological symptoms or disorders.

Ultrahigh magnetic field strengths operating at >3T, offer increased sensitivity and resolution to detect, quantify and characterize minute PVS. Although manual detection and tracing of PVS is extremely time-consuming and subject to inter-reader bias, it remains the current standard for detection and quantification. User-assisted algorithms using Frangi filters have been implemented and proven to reduce tracing time, improve accuracy and detection of PVS in 3 T and 7 T images (33–40). Our aim was to employ Perivascular Space Semi-Automatic Segmentation (PVSSAS) for PVS tracing, which uses a Frangi-based detection algorithm with a user-friendly GUI to aid the speed and ease with which PVS are identified and quantified across the entire brain to better understand the potential neuroinflammatory association with COVID-19. The use of UHF MRI, detection of group differences and correlation of PVS number, volumetric measures, and symptoms may provide insight on how PVS contribute to glymphatic drainage and the COVID-19 disease process.

## Methods

### Subjects

Ten COVID-19 patients between the ages of 18–80 were prospectively recruited at the Mount Sinai Health System through database search and referrals from collaborating physicians and were age and sex matched with nine healthy control participants (Table 1). Institutional Review Board approval was obtained prior to recruitment and written, and informed consent was obtained from all subjects prior to UHF scanning. Eligibility among the patient cohort was confirmed by patients reporting a positive COVID-19 diagnosis and at least one of the following: any neurological symptom such as anosmia or altered taste/smell, brain fog, stroke, encephalopathy, delirium, or memory impairment at the time of COVID-19 diagnosis or following onset of the disease. Patients with a history of agitation, Parkinson's Disorder, apathy, depression, anxiety, hallucinations, and personality disorder were excluded. All participants were screened to assess eligibility for MRI, and subjects with a ferromagnetic or otherwise non-MR compatible implant or device, extreme claustrophobia, or pregnancy were excluded. Control subjects were individuals with no history of COVID-19 and free of any neurological conditions. COVID-19 patient's disease severity was assessed using NIH guidelines, National Institute of Health (NIH) COVID-19 Treatment Guidelines Panel (41) under clinical spectrum (https://www.covid19treatmentguidelines.nih.gov/) along with imaging results, if available.

**Table 1:** Demographic information.

	**COVID-19**	**Control**	***P*-value**
*N*	10	9	
Age mean (SD)	53.6 (9.06)	51.2 (9.15)	0.577
Number of females/males	5F/5M	5F/4M	0.809
BMI (kg/m^2^) mean (SD)	27.94 (5.69)	25.93 (4.34)	0.408
Number of days hospitalized mean (SD)	13.3 (18.07)	N/A	
Number of symptoms mean (SD)	5.8 (4.78)	N/A	
Diabetes (total # of patients)	2	N/A	
Hypertension (total # of patients)	2	N/A	
Smoker (total # of patients)	2	N/A	
Fever (total # of patients)	3	N/A	
Number of patients with mild COVID-19	2	N/A	
Number of patients with moderate COVID-19	4	N/A	
Number of patients with severe COVID-19	2	N/A	
Number of patients with critical COVID-19	2	N/A	
Days between recovery and 7T scan range (median)	50–596 (580)	N/A	

*Participant demographics and accompanying statistical measures. Group differences were assessed for significance using a two-sided T-test. For days between recovery and 7T scan, we calculated 14 days post diagnosis date for non-hospitalized patients, and for patients who were hospitalized, we calculated recovery at time of discharge from the hospital. COVID-19 severity was assessed based on imaging findings, if available, incidence of intubation, receival of supplemental oxygen, etc. along with NIH based clinical spectrum of SARS-COV-2 infection*.

### Image Acquisition

Data were acquired at the Icahn School of Medicine Biomedical Engineering and Imaging Institute (BMEII). Structural and functional MRI data were acquired on a Siemens 7 T Magnetom system scanner (Siemens Healthcare GmbH, Erlangen, Germany) equipped with a SC72CD gradient coil and using a single channel transmitter and a 32-channel receive head coil (Nova Medical, Wilmington, MA). Each subject underwent a 7 T MRI scan which included a T_1_-weighted MP2RAGE wip944 0.7 mm isotropic sequence (42) with generation of Uniform Denoise (“UNIDEN”) images (Acquisition time 8:08; voxel size 0.7 × 0.7 × 0.7 mm^3^; field of view (FOV) 168 × 224 mm^2^; time repetition (TR) 6,000 ms; echo time (TE) 3.62 ms; TI1/TI2 1,050/3,000 ms; flip angle (FA) = 7°; Matrix 320 × 240; bandwidth (BW) 300 Hz/pixel); and an axial T2-weighted turbo spin echo (T2TSE) sequence (minimum TR = 9,000.0 ms, TE = 59 ms, flip angle = 158°, FOV = 168 × 200 mm^2^, matrix = 512 × 432, in-plane resolution 0.2 × 0.2 mm^2^, slice thickness = 2 mm, slice gap = 0.6 mm, slice number = 56, BW = 279 Hz/pixel, minimum time = 6:21 min).

### Image Preprocessing

**Figure 1:**
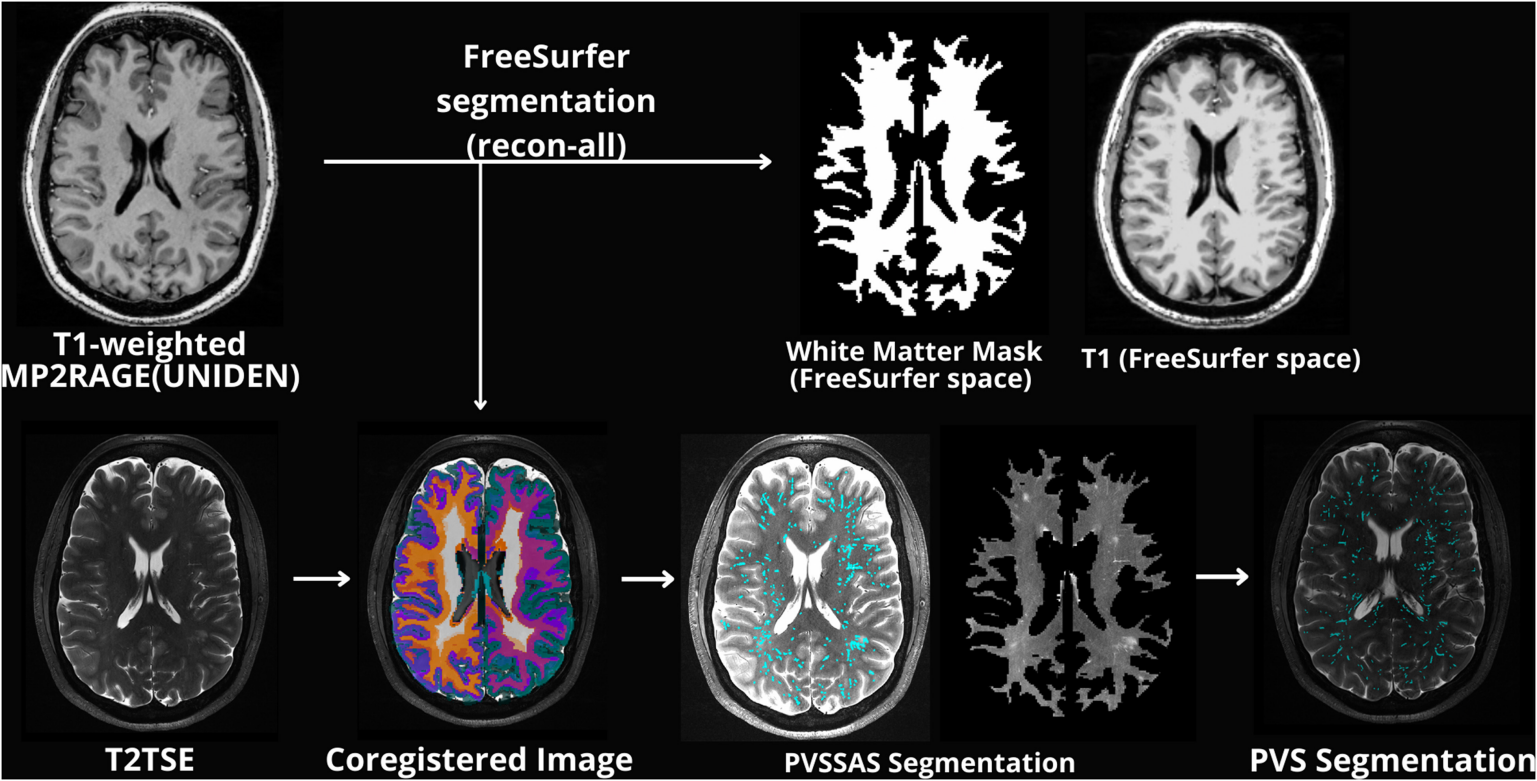
Schematic example of the general preprocessing workflow displaying original structural images along with images processed through PVSSAS. Images labeled PVSSAS segmentation display a T2TSE with PVS marked by PVSSAS along with an unmarked white matter mask of the original T2 image.

Following imaging acquisition, all T_1_-weighted MP2RAGE images were processed using FreeSurfer 7.2 (43, 44) with motion correction, intensity normalization, skull stripping and neck removal, automatic segmentation, and parcellation processing steps. Generated T_1_ weighted image and a white matter mask were appropriately coregistered to the T_2_TSE image using Statistical Parametric Mapping (SPM12, RRID:SCR_007037). The T_2_TSE and co-registered white matter mask were used as inputs into PVSSAS to identify PVS. This workflow is shown in Figure 1.

### PVSSAS, Validation, and Segmentation

**Figure 2:**
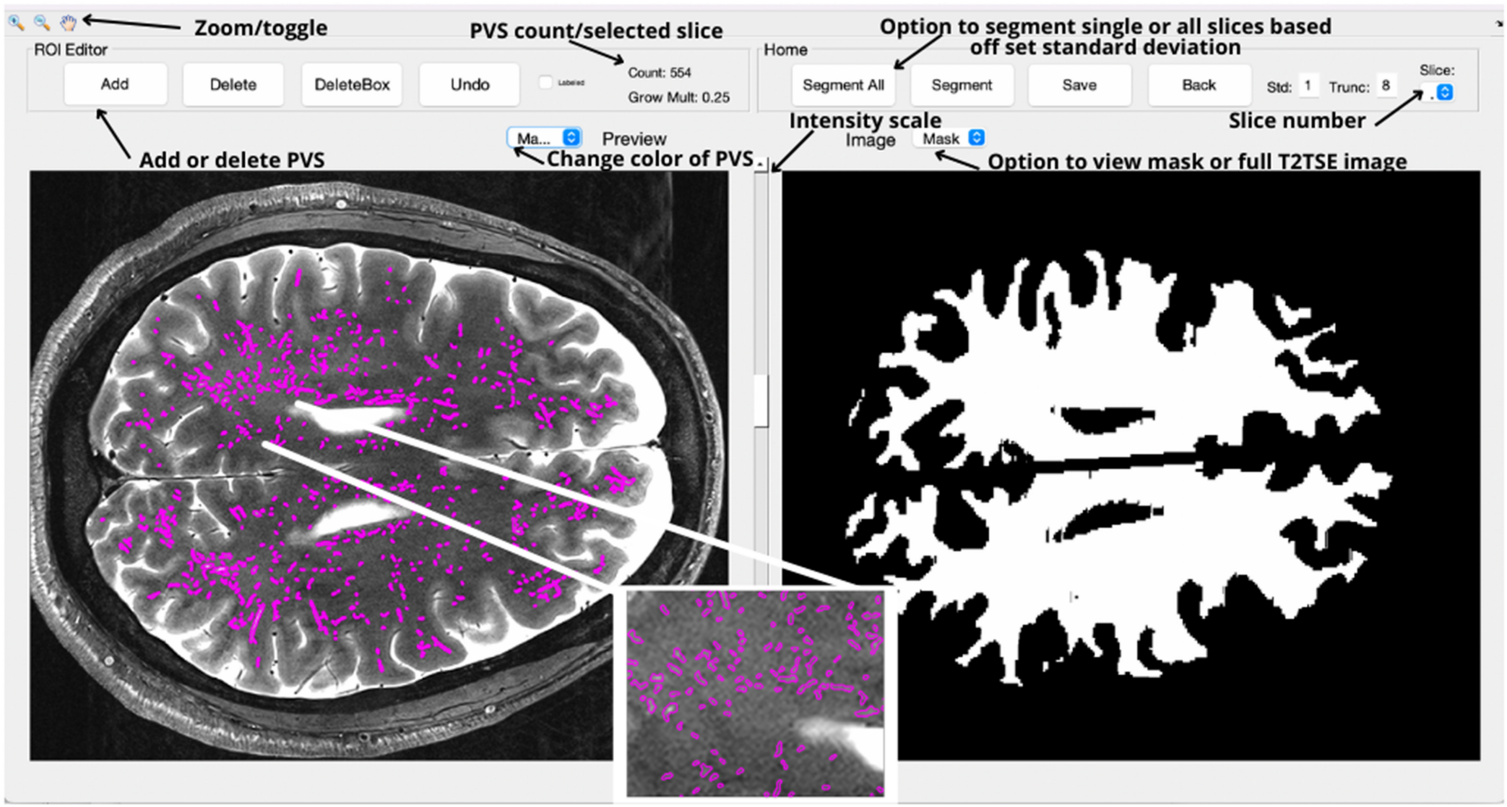
The primary interface for the PVSSAS tool, with images rotated 90 degrees by convention. In the right view panel, the GUI displays white matter mask for the selected slice. On the toolbar, options are available for segmenting the whole brain, the selected slice, saving tracing masks, or for altering the parameters for the segmentation algorithm. In the left view panel, the completed segmentation can be viewed and edited—a trained reader can add or remove tracings.

Visualization and detection of PVS was performed using PVSSAS, a semi-automated tool for segmenting, viewing, and editing PVS in the brain. PVS are preliminarily segmented using a 2D Frangi filter, a quick and powerful segmentation technique which detects vessel-like structures and fibers in 2 and 3 dimensions, such as PVS (Figure 2). Frangi filters have been previously implemented to perform segmentation of PVS at 7T, however, each method may have limitations and with room for improvement (35, 39, 40).

An external Frangi filter package made for Matlab (The MathWorks Inc., Natick, MA) was used (45). Prior to application of the Frangi filter, the PVSSAS program was used to pre-process the T_2_-weighted structural images by applying a Gaussian blurring function and normalization of the voxel intensity. PVSSAS pre-processes the white matter mask by filling in small holes, under 200 voxels in size and performing a morphological erosion to remove potential false positives along the gray-white matter boundary. The Frangi filter is then applied to the input white matter mask region, using an adjustable setting sensitivity parameter (set to 1 standard deviation for the presented data). Additionally, PVS are filtered by size, excluding markings too small or large to be reliably identified or present outside of the white matter mask, such as regions of CSF like ventricles. A range of 4–300 voxels was selected in consultation with radiologists and researchers on the T2-weighted imaging dataset. This avoids the detection of noise artifacts on the small end and portions of the ventricles on the large end. Frangi parameters were chosen among those that produced fair agreement with several manual markings and made to resemble optimal parameters as determined by Ballerini et al. (35).

PVSSAS was validated based on the evaluation of relative speed, accuracy, and sensitivity of both manual and tool-assisted methods. Manual PVS segmentation and marking was originally performed in OsiriX software (Pixmeo SARL, Bernex, Switzerland) by a trained and board-certified Neurologist with over 20 years of experience. PVS were manually marked throughout, requiring ~6 h to mark all 54 brain slices. Manual tracings marked cross-sections rather than boundaries and so couldn't be used to determine axis length or volume, An additional rater was trained to identify PVS on 7T T2TSE scans using PVSSAS, and was instructed to focus on removing erroneous markings, not on adding additional ones. It took ~30 min for the rater to completely mark the scan using PVSSAS. Separately, the inter-rater reliability of the two raters was assessed using PVSSAS, again with the focus on removing erroneous markings, not adding additional ones.

Validation of the PVSSAS tool focused on the quantitative sensitivity and specificity of both methods compared to each other. This step was performed because manual tracings in OsiriX identified each PVS with a cross-sectional point, rather than as volumes. Therefore, it was not possible to reliably compare other useful measures such as the relative length, average volume or total volumes of each. Sensitivity measures were determined by comparing the total number of PVS found using each method. The technique included several inherent constraints: (1) since the voxel thickness and slice gap preclude detection of PVS traversing slices, these spaces were counted as separate PVS in both the semi-automated and manually traced methods; (2) all PVS above 300 voxels in size were unlikely to be PVS, rather likely ventricles or a portion of a ventricle, which due to slice position may be perceived as a PVS and henceforth not marked; (3) PVS outside the white matter mask were excluded, as the semi-automated method was calibrated for detection of white matter PVS only.

Specificity measures were determined by performing a slice-by-slice comparison between the semi-automated and manual tracings. For each slice the centroid (center voxel) of every tracing was determined using the regionprops 3 command in Matlab, and all centroids within 0.7 mm were considered successful overlap between the manual and semi-automated tracings. This number was determined based on an analysis of the number of PVS overlapping relative to the distance to the nearest match. Above ~0.7 mm, the distance to the closest match rapidly increases.

In this study, PVSSAS was implemented in all subjects and automated PVS markings were generated following segmentation with a standard deviation of 1. Each slice was manually inspected for misclassified PVS (those outside the white matter, including within a sulcus or ventricle, or hyperintense structure), and mismarked PVS were removed. Following the inspection of all slices, data were exported as a binary.mat file, with PVS voxels labeled as 1 and all other voxels as 0. To assess inter-rater reliability between readers using the PVSSAS, the Sorensen-Dice coefficient was calculated in MatLab using Sørensen-Dice similarity coefficient.

### Statistical Methods

The PVS were characterized on a multi-slice 2D basis, due to non-isotropic voxels, resulting in the following measures per subject: PVS density (PVS voxel count / white matter volume), PVS count, median PVS volume, total PVS volume, median equivalent diameter, median long axis length, and median short axis length. Statistical data were processed in RStudio (46). Shapiro–Wilk testing was performed to assess for normality due to its performance in small sample sizes (47). Group differences for all PVS measures, total white matter volume, total intracranial volume, and BMI were assessed using two-tailed Student's *t*-test in all cases in which data was normally distributed. In instances where one or both measures were not normally distributed, Wilcoxon Signed Rank was used to assess differences. Statistically significant group differences, such as count, and white matter were performed with correction for total intracranial volume. Pairwise statistical correlations between PVS count, total white matter volume, total intracranial volume, Body Mass Index (BMI), cardiovascular risk factors such as diabetes, smoking, and hypertension, and symptoms were evaluated first using partial correlation or binomial linear regression, without correction. In the COVID-19 patient group, correlations between PVS count and hospital stay duration and number of symptoms was assessed. For all statistically significant correlations, partial correlations were performed with correction for age and gender with binomial linear regression.

## Results

### PVSSAS Validation

**Figure 3:**
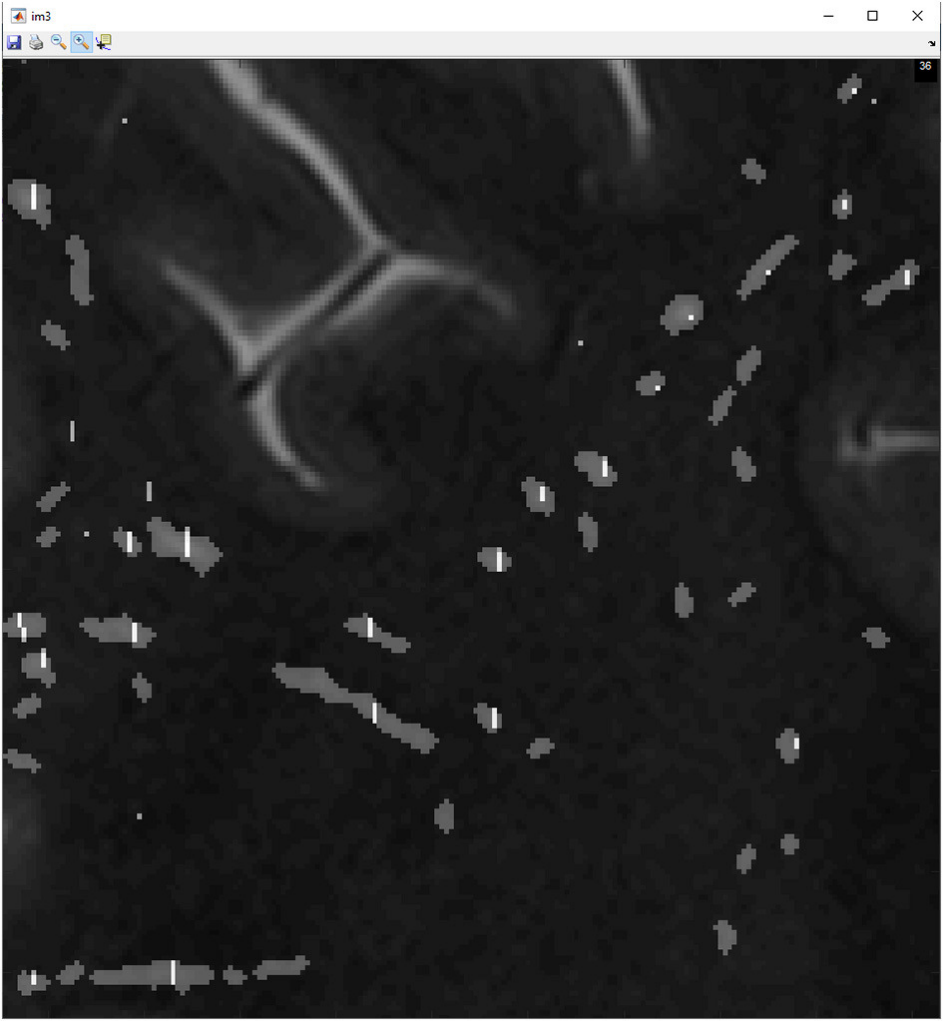
This figure shows manually marked PVS denoted by a white line shown overlapping with gray markings, semi-automated marked PVS by employing the use of PVSSAS.

A total of 2,435 PVS were identified by the semi-automated method, and 3,743 PVS were manually identified within the white matter mask. A tolerance of 0.7 mm was used, resulting in 83% of all PVS detected by the semi-automated method which matched with the manual dataset and 94% of the manual PVS matched within the semi-automated dataset. As shown in Figure 3, there was generally excellent agreement between the manual and semi-automated markings in any given slice. The inter-rater reliability between the two readers using the semi-automated method was as follows: of the PVS identified by reader 1, 97.8% of the PVS were in the identical location as reader 2, and 99.6% were within 0.8 cm. Of the PVS identified by reader 2, 99.2% were in the same location as reader 1, and 99.9% were within 0.8 cm. Reader 1 identified 1,680 PVSs, and reader 2 identified 1,656 PVS. The Dice score between the two readers segmentations was 0.9914.

### Group Differences

**Figure 4:**
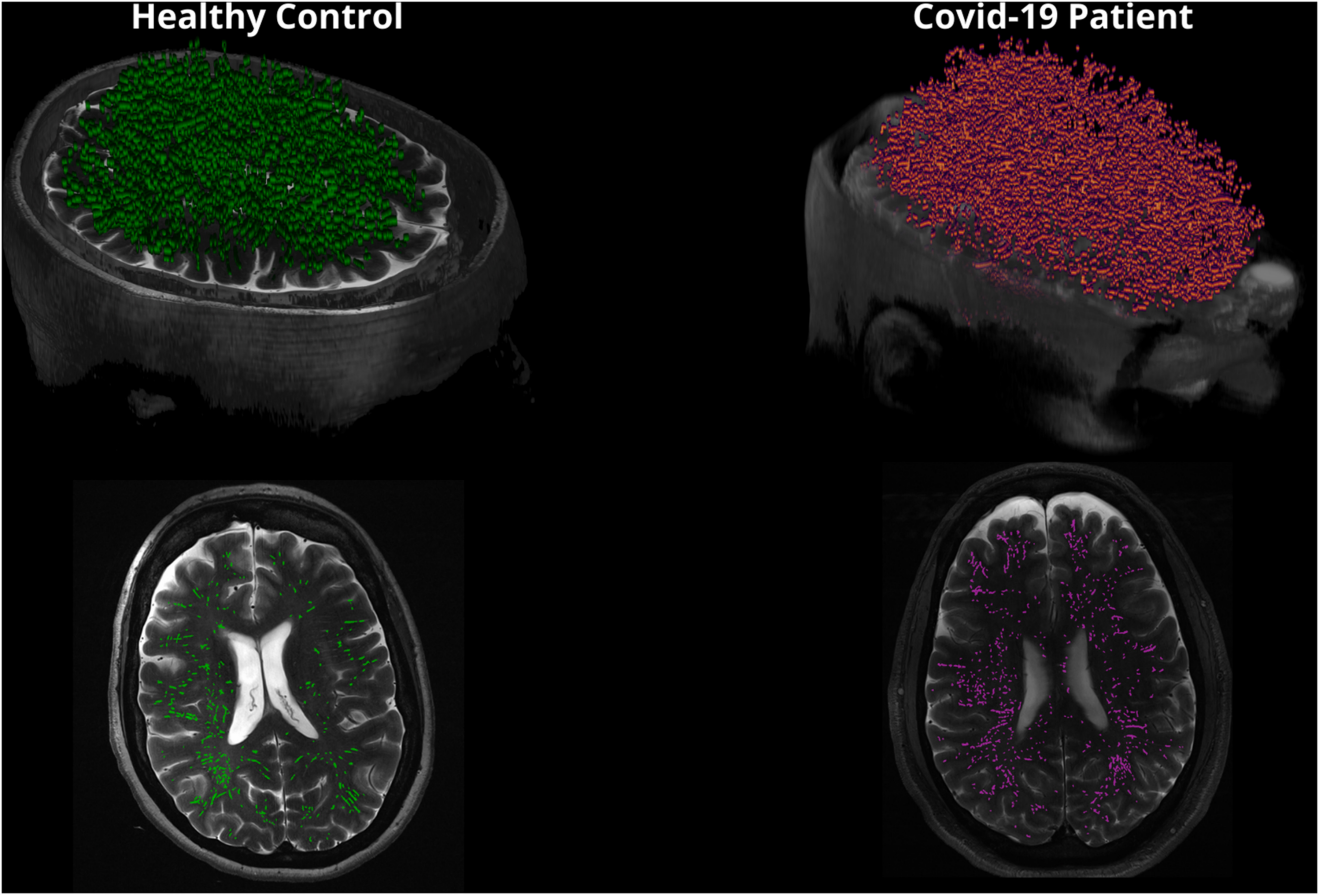
This figure is a 3D rendering of PVS in healthy controls compared to COVID-19 patients. Note that PVS are magnified in the 3D rendering for visualization purposes. The bottom images reflect a single slice in the axial view displaying PVS.

The PVS measures and volumetrics in the COVID-19 patients and healthy controls are summarized in Table 2. This study demonstrated that the PVS count was significantly higher in COVID-19 patients compared to healthy controls (*p* = 0.0373) and total white matter volume was significantly increased in COVID-19 patients compared to healthy controls (*p* = 0.0435). Figure 4 shows a 3D rendering to visualize differences in PVS count in healthy controls compared to COVID-19 patients. There was no statistically significant difference between other PVS measures or intracranial volume metrics between groups (Table 2).

**Table 2:** PVS Measures and volumetrics in COVID-19 compared to healthy controls.

**Measure**	**COVID-19**	**Healthy controls**	***p-*value**
***N***	**10**	**9**	
Median volume mean (SD), mm^3^	26.2 (2.49)	25.6 (1.74)	0.501
Count mean (SD)	3928 (866)	3,232 (350.5)	0.0373^*^
Total volume mean (SD), mm^3^	246458.1 (71696)	217960.33 (58,743)	0.296
Density mean (SD), PVS/mm^3^	2.30 (0.51)	2.34 (0.57)	0.746
Median Eq. distance [mean (SD)], mm	3.68 (0.11)	3.65 (0.083)	0.511
Median long axis mean (SD), mm	8.09 (0.53)	8.04 (0.36)	0.875
Median short axis mean (SD), mm	4.29 (0.12)	4.22 (0.07)	0.178
White matter volume mean (SD), mm^3^	477,065 (62,279)	411,236 (34,072)	0.0435^*^
Total intracranial volume mean (SD), mm^3^	1,263,358 (204,449)	1,345,119 (108,410)	0.107

*Average PVS measures and volumetrics on a whole-brain basis for both healthy controls and COVID-19 patients. All PVS measures besides count are represented in voxels. Reported corrected p-value is corrected for age and sex*.

* *Denotes statistically significant p-values*.

*Bold values indicates statistically significant finding*.

### Symptom Measures

**Figure 5:**
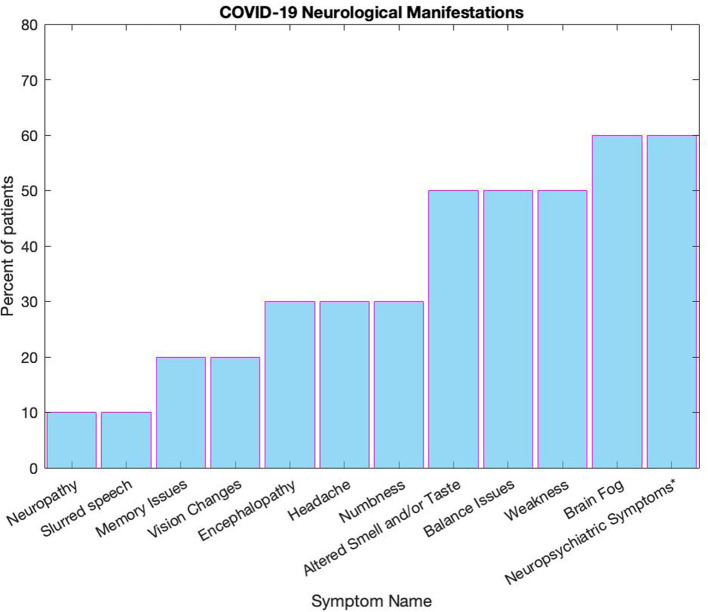
This figure displays the range of symptoms reported by patients, all numbers are reported in percentages. Some symptoms were grouped together, such as neuropsychiatric symptoms* which includes personality changes, anxiety, depression, and insomnia Altered smell and/or taste includes loss of smell or taste, dysgeusia, and anosmia, and vision changes included both loss of vision and double vision. Balance issues were defined by issues with balance as well as if a fall was recorded, and confusion was grouped with encephalopathy.

Assessment for presence of COVID-19 symptoms in the 10 patients revealed 30 symptoms (Figure 5). In addition, three patients reported being intubated or receiving supplemental oxygen at home. This study, although limited in the number of patients, demonstrated a significant association between white matter volume and self-reported brain fog (*p* = 0.035). There was no significant correlation between other neuropsychiatric manifestations, such as personality changes, anxiety, depression, and insomnia and PVS count, white matter volume, or with total intracranial volume.

### Relationship Between Other Disease Severity Factors

An assessment was made between PVS count, total white matter volume, and intracranial volume with markers of disease severity (hospital admission duration and total number of symptoms) and incidence of comorbidities such as obesity (i.e., BMI) (Table 3). There was no correlation between PVS count, total white matter volume, total intracranial volume, BMI, number reported symptoms, or hospitalization duration. Additionally, when looking at cardiovascular risk factors such as diabetes, smoking, and hypertension, which may affect PVS count, we found no correlation among any of the factors. There was a significant correlation between PVS count and white matter volume in COVID-19 patients (Correlation coefficient = +0.829, *p* = 0.003), controls (Correlation coefficient = 0.769, *p* = 0.016) and within all subjects (Correlation coefficient = +0.86, *p* = 2.38 × 10^−6^). Additionally, there was a significant correlation between PVS count and BMI within the patient group (Correlation coefficient = +0.849, *p* = 0.002) and within all subjects (Correlation coefficient = +0.633, *p* = 0.005), however there was no correlation within the control group. There was a correlation between BMI and total white matter within the patient group (Correlation coefficient = +0.702, *p* = 0.024), and within all subjects (Correlation coefficient = +0.579, *p* = 0.012), and no correlation within the control group. There were no correlations between total intracranial volume and PVS count or BMI in any group.

**Table 3:** Correlation measures.

	**COVID-19 Correlation coefficient (*p*-value)**	**Control Correlation coefficient (*p*-value)**	**All subjects Correlation coefficient (*p*-value)**
PVS count: white matter volume	**+0.829 (0.003)**	**+0.769 (0.016)**	**+0.860 (2.38 × 10** ^**−6**^ **)**
PVS count: total intracranial volume	+0.470 (0.170)	+0.288 (0.4529)	+0.253 (0.296)
PVS count: BMI	**+0.849 (0.002)**	**–**0.204 (0.6278)	**+0.633 (0.005)**
White matter volume: BMI	**+0.702 (0.024)**	+0.175 (0.6794)	**+0.579 (0.012)**
Total Intracranial Volume: BMI	+0.317 (0.371)	+0.238 (0.5704)	+0.210 (0.403)
PVS count: hospitalization duration (days)	+0.190 (0.600)	N/A	N/A
PVS count: number of symptoms	−0.334 (0.346)	N/A	N/A

*Association between PVS count, total white matter volume, total intracranial volume, and BMI*.

*Bold values indicates statistically significant finding*.

## Discussion

In the present study, a workflow was established for detection of PVS at UHF, providing a feasible tool to extract a possible non-invasive imaging marker in a preliminary investigation in COVID-19 patients with documented neurologic impairment. This study assessed the detection of PVS and association with neurological symptoms and comorbid markers that affect disease severity, such as BMI, in COVID-19 patients which may be associated with a cascade associated with the process neuroinflammation. The 7 T MRI neuroimaging data found observable neuropathological differences between patients with COVID-19 and healthy controls. Although there was no significant association to report between the total number of PVS and neurological symptoms, intubation, or hospitalization, this is one of the first preliminary studies evaluating the role of PVS in COVID-19 patients with neurologic impairment. Analysis of group differences within PVS count and white matter volume and correlation between these measures and BMI resulted in interesting findings. This preliminary study demonstrated a statistically significant difference between PVS count and overall white matter volume between healthy controls and COVID-19 patients. The data also demonstrated a significant correlation between PVS count and BMI, and white matter volume and BMI within the COVID-19 patient cohort and within all subjects. Interestingly, in this study there was a significant correlation between white matter volume and self-reported brain fog, although a significant correlation was not found between PVS and this symptom. Although a larger study is needed to fully ascertain this relationship, this difference may indicate that these two metrics serve as markers for separate neuroinflammatory effects on the brain or possible involvement in processes associated with neuroinflammation.

It is believed that COVID-19 patients may develop neuroinflammation, and many of the diverse long-term neurological symptoms in COVID-19 may also relate to neuropathology occurring from neuroinflammatory responses, which are distinct from ischemic events secondary to altered coagulation (6). In this preliminary study comparing COVID-19 patients with neurologic symptoms with age and sex matched controls using ultra-high field 7 T MRI, the COVID-19 patients demonstrated increases in PVS count and white matter volumes. These data suggest that PVS may serve as a potential neuroimaging biomarker of neurological manifestations in COVID-19 patients. Further, we found a strong positive correlation between increased numbers of PVS and total white matter volume. This may be related to white matter volume changes expanding the apparent volume with the differences related to the consequences of inflammatory cellular infiltration and fluid, rather than increasing native cellular volume or could point to a possible connection to a neuroinflammatory response. In other areas of research, an increase in white matter volume has also been linked to neuroinflammatory processes, such as in aging and disorders such as Alzheimer's disease (48). Indeed, our data demonstrated a significant correlation between white matter volume and self-reported post-COVID-19 brain fog, suggesting that neuroinflammation may be contributory to post-COVID-19 cognitive impairment. While we did not find a relationship between brain fog and PVS, previous larger studies have found associations between PVS and decreased cognitive performance in older adults (49) suggesting an area for future study.

In the present study, the increase in white matter volume within the COVID-19 group compared to controls further indicates that the COVID disease process may have a neuroinflammatory component. Strong associations between increased white matter volume and increased PVS count may further enhance the link between PVS count and neuroinflammation. This indicates that PVS count may be a viable marker for neuroinflammation or may precede neuroinflammatory processes in other diseases aside from COVID-19.

Currently, published research demonstrates how comorbidities, including diabetes and obesity, can lead to increased COVID-19 severity (50–53). Correlation measures of neuroinflammatory markers such as PVS count and white matter volume with BMI and potentially glucose levels may be indicative of increased likelihood of neurologic sequelae from COVID-19. If neuroinflammatory coagulopathic events lead to disruption of the glymphatic clearance in the central nervous system in COVID-19, this in turn may lead to an increased size and number of PVS, as demonstrated in this cohort of patients. More specifically, glymphatic dysfunction may exacerbate or trigger neuroinflammation (54), leading to cognitive dysfunction, a growing phenomenon among COVID-19 survivors, who are at high risk of developing cognitive impairments (55).

This study using ultra-high field 7 T MRI is one of the first to also implement a semiautomated PVS detection tool to quantify PVS in COVID-19 and establish a mechanism to probe underlying neurological changes potentially involved in neuroinflammatory processes linked to COVID-19. Importantly, in this study all age and sex matched healthy control subjects were scanned prior to the pandemic as part of a broader UHF neuroimaging initiative, mitigating the possibility of inadvertently including an asymptomatic COVID-19 patient in the control group, and providing confidence in the validity of the control group compared to the COVID-19 patient cohort.

This study has several limitations. First, given the preliminary nature of this study, we were limited to a small sample size of COVID-19 patients with neurologic symptoms. Although there were no significant results at the *a priori* threshold level, the data suggest that correlations may trend toward significance with a larger patient cohort. Thus, future studies with a more robust sample size may have greater power to detect such differences and to control for confounding factors that may provide an improved understanding of how the size and number of PVS correlate to neurological symptoms. A second limitation of the present study is the lack of a symptom severity score, which could assist with determining overall disease severity beyond treatment, imaging, and hospital duration and limiting the ability to associate possible inflammatory markers with incidence of severe disease. Future studies with more granular classification may provide better insight into disease progression and severity. Another limitation of the present study is that the current patient cohort scanned at 7 T did not have a baseline MRI prior to having COVID-19, limiting the ability to compare volumetrics and PVS count pre- and post-infection. We also had limited access to medical records containing pertinent information (e.g., bloodwork to identify inflammatory markers at time of infection) and did not collect blood at time of 7 T scan acquisition, limiting our ability to determine a relationship between neuroimaging findings and specific inflammatory markers. Future studies should investigate the link between pro-inflammatory mediators such as interleukin- (IL)-1β, tumor necrosis factor (TNF)-α, and C-reactive protein to better understand the link between PVS and inflammatory response. While PVSSAS is a strong tool to reduce manual detection of PVS, it should be noted that the T2 images used to identify PVS were acquired with a 2D T2-weighted TSE sequence. Our 7 T scanner is equipped with the SPACE sequence (Sampling Perfection with Application optimized Contrasts using different flip angle Evolution), which is a 3D T2-weighted sequence. However, we preferred to use the 2D TSE sequence for this application because in our experience, the image contrast in the 2D TSE sequence is more uniform over the imaged region than the SPACE sequence. Future studies will focus on developing optimized contrast for 3D isotropic T2-weighted sequences as well as tools to investigate morphological changes of PVS using 3D acquisitions. Future studies with a larger patient cohort should incorporate symptom severity measures and, if possible, include patients with MRI scans pre- and post-COVID-19 along with inclusion of inflammatory markers through bloodwork. This may help to corroborate and elaborate the current findings, and the efficacy of using PVS as a potential neuroimaging biomarker.

## Conclusion

This study on COVID-19 patients with neurologic sequelae demonstrated an increase in size and number of PVS compared to an age- and sex-matched cohort of patients. As the time course for PVS development is not well-established, presence of some PVS may predate COVID-19 infection, or be a consequence of disease, indicating that PVS could be a risk factor for symptomatic consequences of COVID-19. Potential hypotheses that patients with more PVS are predisposed to the effects of COVID-19 due to limitations in underlying responses to inflammation, resulting in exacerbation of COVID-19 symptoms and possible predisposition to development of long-haul syndrome. These preliminary findings provide an indication that symptomatic neurological manifestations of COVID-19 may be related to alterations within the glymphatic clearance system that may be caused by and contribute to a neuroinflammatory response. The ability to rapidly and accurately evaluate size and number of PVS on MRI, may be potentially applied to additional neurologic disorders, and serve as a useful method for assessing COVID-19 patients with neurologic symptoms as well help elucidate the neuroinflammatory neuropathologic mechanisms as more evidence emerges.

## Data Availability Statement

The raw data supporting the conclusions of this article will be made available by the authors, without undue reservation.

## Ethics Statement

The studies involving human participants were reviewed and approved by Institutional Review Board, Icahn School of Medicine at Mount Sinai. The patients/participants provided their written informed consent to participate in this study.

## Author Contributions

ML: writing and editing of the manuscript as well as data collection and analysis, figure preparation, and review of final submission. GV, DS, MM, and LM: development of PVSSAS tool for analysis. DR, DS, and GV: validation of PVSSAS tool. SR, OK, BM, SS, and JG: management of recruitment and collection of data. OK and PBa: overall covid neuroimaging study design. NJ: characterization of neurological symptoms and patient recruitment. BD and PBe: characterization of neuroimaging abnormalities. BD: overall direction of study design. PBa, BD, CK, and JB: data interpretation, writing, and editing the manuscript. PBa is the principal investigator of the overall neuroimaging study. All authors contributed to writing and editing of the manuscript and review of final submission.

## Funding

This work was supported by NIH grants; R21NS122389 and R01CA202911. NJ is the Icahn School of Medicine at Mount Sinai Bludhorn Professor of International Medicine.

## Conflict of Interest

PBa is a named inventor on patents relating to magnetic resonance imaging (MRI) and RF pulse design. The patents have been licensed to GE Healthcare, Siemens AG, and Philips international. NJ receives grant funding paid to her institution for grants unrelated to this work from NINDS (NIH U24NS107201, NIH IU54NS100064, NIH U24NS113849). She receives an honorarium for her work as an Associate Editor of *Epilepsia*. The remaining authors declare that the research was conducted in the absence of any commercial or financial relationships that could be construed as a potential conflict of interest.

## Publisher's Note

All claims expressed in this article are solely those of the authors and do not necessarily represent those of their affiliated organizations, or those of the publisher, the editors and the reviewers. Any product that may be evaluated in this article, or claim that may be made by its manufacturer, is not guaranteed or endorsed by the publisher.
